# A Proof-of-Concept Framework for Upper-Limb Segment Definition and Joint Angle Computation Using Marker-Based Motion Capture

**DOI:** 10.3390/s26165047

**Published:** 2026-08-09

**Authors:** Catarina M. Amaro, Hannah Rice, Maria António Castro, Rui Mendes, Beatriz B. Gomes

**Affiliations:** 1 Interdisciplinary Center for Human Performance (CIPER), Faculty of Sports Science and Physical Education, University of Coimbra, 3040-256 Coimbra, Portugal; beatrizgomes@fcdef.uc.pt; 2 Centre of Mechanical Engineering, Materials and Processes (CEMMPRE—ARISE), Department of Mechanical Engineering, University of Coimbra, 3040-248 Coimbra, Portugal; maria.castro@ipleiria.pt; 3 Department of Physical Performance, Norwegian School of Sport Sciences, Sognsveien 220, 0863 Oslo, Norway; hannahr@nih.no; 4 School of Health Sciences, ciTechCare, CDRSP, Polytechnic University of Leiria, 2411-901 Leiria, Portugal; 5 RoboCorp Laboratory, i2a, IPC Polytechnic Institute of Coimbra, 3045-093 Coimbra, Portugal; rmendes@esec.pt; 6 Polytechnic Institute of Coimbra, Coimbra Education School, 3030-329 Coimbra, Portugal; 7 Sport Physical Activity and Health Research & Innovation Center (SPRINT), 3030-329 Coimbra, Portugal

**Keywords:** motion capture, biomechanics, upper limb, joint kinematics, segment definition, marker-based analysis

## Abstract

Marker-based motion capture systems are widely used to estimate joint kinematics, yet their accuracy depends strongly on how anatomical segments and coordinate systems are defined. This proof-of-concept study aimed to describe and technically evaluate a structured and reproducible framework for upper-limb segment definition and joint-angle computation. Reflective markers were placed on anatomical landmarks of the trunk and upper limbs, and joint angles were computed using custom-developed MATLAB R2022b (MathWorks, Natick, MA, USA) routines. Baseline-corrected model-derived joint angles were compared with composite reference measurements obtained using a universal manual goniometer and a twin-axis biosignalsplux goniometer under predefined static conditions in two adult participants. Side-specific mean absolute error values ranged from 0.80° to 5.24°, while root mean square error values ranged from 0.86° to 5.24°. The largest discrepancies were observed during maximum wrist extension and left maximum radial deviation. The evaluated static observations showed close correspondence in several joint positions, although larger discrepancies occurred in selected end-range wrist positions. Given the limited sample, single recordings, and controlled static conditions, these findings should be interpreted as an initial demonstration of technical feasibility rather than evidence of generalisable validity or repeatability. The explicit framework provides a basis for further evaluation using larger samples, repeated marker applications, repeated trials, and dynamic multi-planar upper-limb tasks.

## 1. Introduction

Marker-based motion capture systems are widely used in biomechanics to estimate three-dimensional joint kinematics in both clinical and sport contexts [1,2,3]. Although markerless approaches have gained increasing attention in recent years, marker-based systems continue to be used as reference methods for validating upper-extremity kinematics under controlled laboratory conditions [4,5].

Despite their widespread use, marker-based motion capture analyses depend strongly on how anatomical landmarks, joint centres, segment coordinate systems, and rotation sequences are defined. However, these methodological choices are not always reported in sufficient detail, which can limit the reproducibility and comparability of upper-limb kinematic analyses.

This lack of standardisation can lead to inconsistencies in joint angle estimation, limiting the comparability of results between studies and reducing the reproducibility of biomechanical analyses. Small differences in marker placement, segment definition, or axis construction may result in meaningful variations in calculated joint kinematics [6,7,8].

In addition to methodological variability, soft tissue artefacts represent a major source of error in marker-based motion capture systems. The movement of skin-mounted markers relative to the underlying bone can introduce significant inaccuracies in the estimation of segment orientation and joint kinematics, particularly when marker placement, anatomical landmark identification, and segment definition are not consistently controlled [6,7,8,9]. These effects are particularly relevant in distal segments and in movements involving larger ranges of motion.

Several methodological frameworks have been proposed to address these challenges, including recommendations from the International Society of Biomechanics (ISB) for joint coordinate systems. For the present study, the ISB recommendations for the shoulder, elbow, wrist and hand are particularly relevant, as they provide standardised definitions for reporting upper-limb joint motion [10]. However, despite their widespread use, there is considerable variability in how segments, joint centres, and coordinate systems are defined across studies [9,10]. This remains particularly relevant in upper-limb analysis, where differences in anatomical landmark identification and segment coordinate system definition can influence the resulting joint angles.

Existing approaches differ in their primary purpose, level of methodological transparency, and adaptability. The ISB recommendations provide standardised anatomical terminology and joint coordinate-system definitions, but they do not prescribe a complete acquisition and processing workflow for every experimental application. Commercial models, such as Plug-in Gait, provide established and automated processing pipelines, although their internal implementation and flexibility may be limited when researchers require custom marker configurations or task-specific calculations. The present framework does not seek to replace these approaches or introduce a new anatomical convention. Instead, it translates established biomechanical principles into a fully explicit and customisable workflow that integrates marker identification, joint-centre estimation, segment coordinate-system construction, bilateral sign harmonisation, trajectory reconstruction, filtering, baseline definition, and joint-angle computation. Its contribution therefore lies in making the complete analytical chain transparent, traceable, and reproducible within custom motion-analysis environments.

To support the technical feasibility of the proposed methodology, model-derived joint angles were compared with measurements obtained using manual and digital goniometers under controlled static conditions, as goniometric and inclinometer-based measurements are commonly used reference procedures for assessing joint range of motion [11,12,13,14].

The present proof-of-concept study aimed to describe and technically evaluate a structured and reproducible framework for upper-limb segment definition and joint-angle computation using marker-based motion-capture data. Specifically, the study examined the correspondence between model-derived joint angles and goniometric reference measurements under controlled static conditions. By combining methodological transparency with a controlled technical evaluation, this study provides a reproducible framework for future biomechanical analyses of upper-limb motion.

## 2. Materials and Methods

### 2.1. Participants and Experimental Setup

Two adult participants were included in this proof-of-concept methodological study. Before data collection, participants were informed about the experimental procedures and familiarised with the static positions to be performed. They were instructed to begin from the anatomical reference position and to maintain each requested posture as steadily as possible for approximately 5 s while the motion-capture and goniometric measurements were obtained. Written informed consent was obtained from both participants before data collection.

Participant 1 was a 30-year-old male basketball player competing at senior level, with a height of 1.80 m, a body mass of 85 kg, and a body mass index of 26.2 kg·m^−2^. He reported five basketball training sessions per week. Participant 2 was a 32-year-old female professional basketball player, with a height of 1.75 m, a body mass of 66 kg, and a body mass index of 21.6 kg·m^−2^. She reported eight basketball training sessions per week. Both participants reported no current upper-limb injury, pain, or condition that limited the execution of the required static positions. The participants were included to support a controlled proof-of-concept evaluation of the complete computational workflow. Reflective markers were placed on anatomical landmarks of the trunk and upper limbs to enable the estimation of shoulder, elbow, and wrist joint angles.

Three-dimensional marker trajectories were collected using a 10-camera Qualisys optical motion capture system (Qualisys AB, Gothenburg, Sweden) at a sampling frequency of 200 Hz. Joint angles were assessed using two reference instruments: a universal manual plastic goniometer with 1° scale graduations, and a twin-axis biosignalsplux Goniometer sensor (GON; PLUX Wireless Biosignals S.A., Lisbon, Portugal), connected to a biosignalsplux acquisition unit. The biosignalsplux sensor has a measurement range of ±150°, a manufacturer-reported accuracy of ±2°, and a repeatability of ±1°. The manual goniometer was used to position the participant and confirm the target joint configuration. The manual and biosignalsplux goniometers then provided two reference measurements of the maintained static position. The reference instruments were positioned to minimise obstruction of the reflective markers, and marker visibility was monitored during acquisition.

Participants performed a series of predefined static upper-limb positions representing anatomical reference and target configurations of the shoulder, elbow, and wrist. Each trial began with the participant standing in the anatomical reference position, with the trunk upright, the upper limbs positioned alongside the body, the elbows extended, and the forearms and hands maintained in a neutral orientation. The same verbal instructions and anatomical alignment criteria were used for both participants.

For the target positions, participants were instructed to move the relevant upper-limb segment to the requested configuration and to avoid visible compensatory movements of the trunk or contralateral limb. Target positions nominally corresponding to 90° were established and checked using the manual goniometer. Maximum positions corresponded to the participant’s greatest actively attained and comfortably maintained range in the requested movement direction.

Each position was performed once by each participant and maintained for approximately 5 s. Participants were instructed to remain as still as possible while manual and digital goniometric measurements and motion-capture data were obtained. The central stable portion of each trial was subsequently used for analysis, excluding the transition frames associated with moving into and out of the target posture. An additional static calibration trial was collected when required to support reference-frame definition and marker reconstruction.

The study was conducted in accordance with the Declaration of Helsinki and was approved by the Ethics Committee of the Faculty of Sport Sciences and Physical Education of the University of Coimbra, as part of the research project in which the present study was included (CE/FCDEF-UC/00812021).

### 2.2. Marker Set

**Figure 1:**
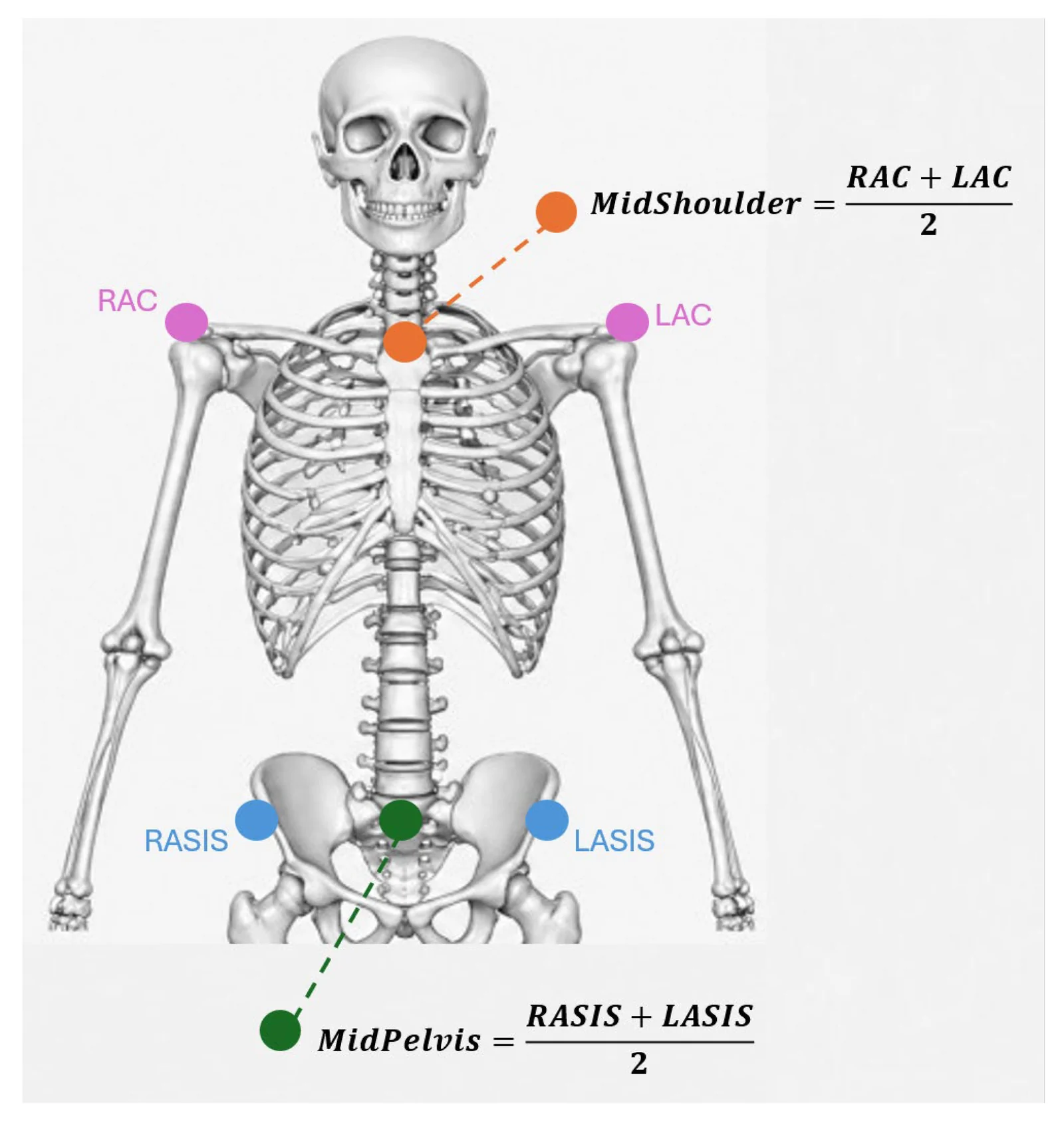
Anatomical placement of reflective markers on the trunk and pelvis. Markers were positioned on the right and left acromion (RAC, LAC) and anterior superior iliac spines (RASIS, LASIS). Midpoints were computed to define the shoulder and pelvis reference points: MidShoulder = (RAC + LAC)/2 and MidPelvis = (RASIS + LASIS)/2.

**Figure 2:**
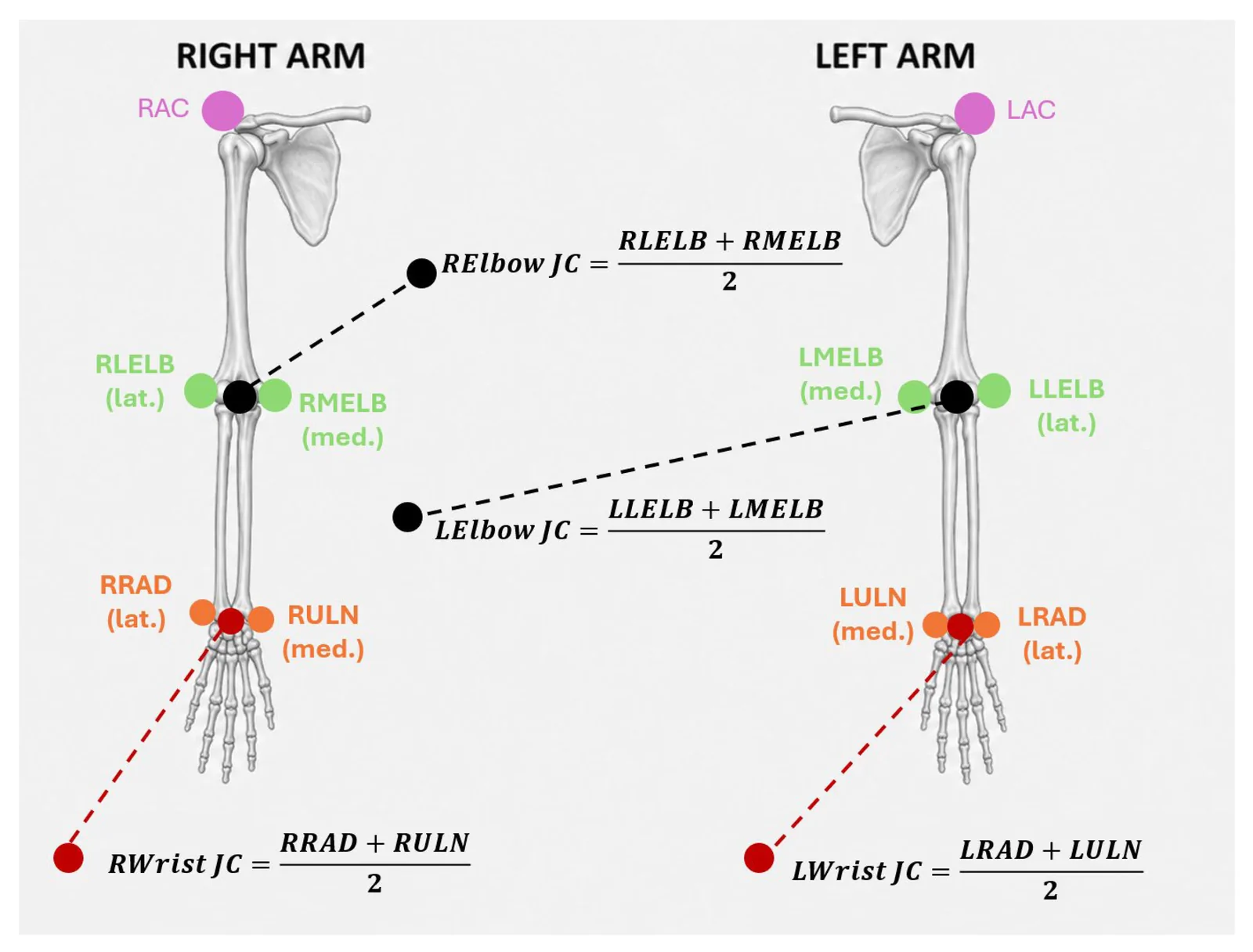
Marker configuration for the upper limb segments (arm and forearm). Reflective markers were placed on the lateral and medial epicondyles of the humerus (RLELB, RMELB; LLELB, LMELB) and on the radial and ulnar styloid processes (RRAD, RULN; LRAD, LULN). Elbow and wrist joint centres were defined as the midpoints between anatomical landmarks: Elbow JC = (lateral + medial epicondyle)/2 and Wrist JC = (radial + ulnar styloid)/2.

**Figure 3:**
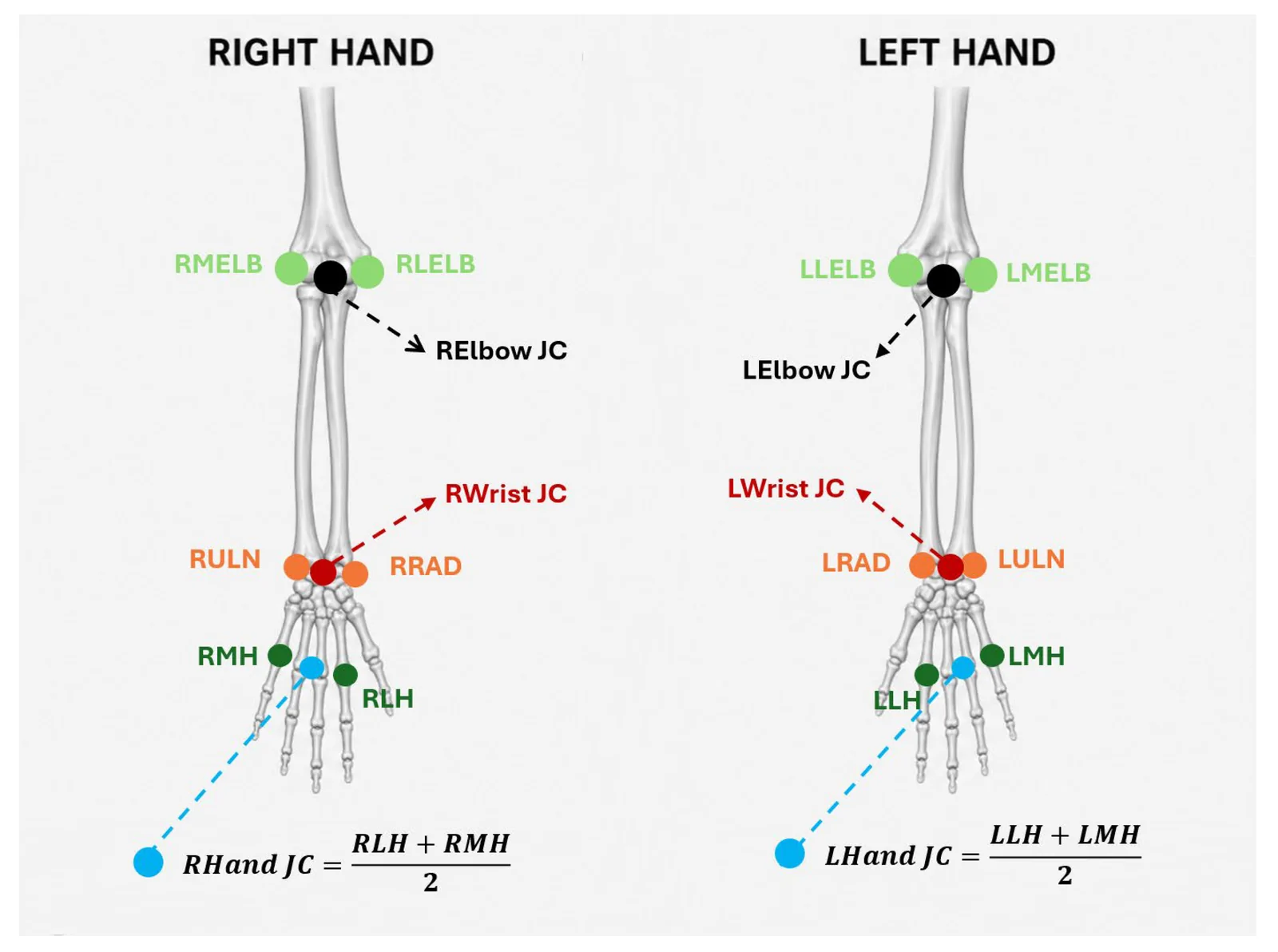
Marker placement for the hand segment. Reflective markers were positioned on the lateral and medial aspects of the hand (RLH, RMH; LLH, LMH). The hand joint centre was calculated as the midpoint between these markers: Hand JC = (lateral + medial hand markers)/2. Wrist joint centres are also shown for reference.

Reflective markers of identical size were placed over palpable anatomical landmarks of the trunk, upper limbs, and hands, as illustrated in Figure 1, Figure 2 and Figure 3. The laboratory setup followed an anatomically based modelling approach informed by the IOR marker model described by [15] and by the recommendations of the International Society of Biomechanics for upper-limb joint coordinate systems [10].

The original IOR protocol was developed primarily for three-dimensional gait analysis and was not adopted as a complete upper-limb model. The present framework retained its general anatomically based principle of using palpable landmarks and bilateral trunk and pelvic reference markers. Specifically, markers placed over the right and left acromia and anterior superior iliac spines were used to define trunk-related reference points.

The upper-limb implementation was developed specifically for the present framework. It included bilateral markers over the medial and lateral epicondyles of the humerus, the radial and ulnar styloid processes, and the lateral and medial aspects of the hands. It also incorporated midpoint-based definitions of the elbow, wrist, and hand reference points; custom local coordinate systems for the trunk, upper arm, forearm, and hand; bilateral sign harmonisation; segment-informed reconstruction of missing paired markers; participant- and side-specific angular baseline definition; and custom MATLAB routines for marker identification, filtering, and joint-angle computation.

All markers were placed by the same trained researcher using the same anatomical palpation and placement procedure for both participants, thereby limiting variability attributable to differences between examiners.

For the trunk (Figure 1), markers were positioned on the acromion processes (RAC, LAC) and anterior superior iliac spines (RASIS, LASIS). Two reference points were defined: the shoulder midpoint (MidShoulder = (RAC + LAC)/2) and the pelvic midpoint (MidPelvis = (RASIS + LASIS)/2).

For the upper limb segments (Figure 2), markers were placed on the lateral and medial epicondyles of the humerus (RLELB, RMELB, LLELB, LMELB) and on the radial and ulnar styloid processes (RRAD, RULN, LRAD, LULN). Elbow and wrist joint centres were defined as the midpoints between paired anatomical landmarks: Elbow JC = (lateral + medial epicondyles)/2 and Wrist JC = (radial + ulnar styloid)/2.

For the hand segment (Figure 3), markers were positioned on the lateral and medial aspects of the hand (RLH, RMH, LLH, LMH). The hand joint centre was defined as the midpoint between these markers: Hand JC = (lateral + medial hand markers)/2.

### 2.3. Data Processing

Marker trajectories were exported from Qualisys in .mat format and processed using custom-developed MATLAB routines. Marker labels were automatically identified using an alias-based matching procedure, and coordinates were converted from millimeters to meters.

Missing marker trajectories were handled using a staged, segment-informed procedure. First, frames were considered eligible for coordinate interpolation when at least three markers within the corresponding marker group were simultaneously and fully available. For these eligible frames, missing coordinates were estimated using linear interpolation.

Second, an anatomical pair-reconstruction procedure was applied to the medial and lateral epicondyle markers of the humerus, the radial and ulnar styloid markers, and the lateral and medial hand markers. For each anatomical pair, a robust mediolateral vector was estimated from frames in which both markers were simultaneously visible. When only one marker of the pair was available in a given frame, the position of the missing partner was reconstructed using the available marker and the corresponding mediolateral vector. The vector was updated from valid trajectory information and, when required, supported by the static reference acquisition.

After the segment-specific interpolation and pair-reconstruction procedures, any remaining missing coordinate samples were filled, where required, using linear interpolation followed by nearest-neighbour filling before filtering. This final step was used to obtain continuous trajectories for signal processing and was not the primary procedure used to define the spatial relationship between paired anatomical landmarks. Previous studies have described interpolation and segment-informed reconstruction strategies for addressing missing motion-capture trajectories, although the suitability of each approach depends on gap location, duration, marker configuration, and movement characteristics [16,17,18].

All marker trajectories were filtered using a fourth-order low-pass Butterworth filter with a cut-off frequency of 12 Hz. Joint angle time series were further smoothed using a fourth-order Butterworth filter with a cut-off frequency of 8 Hz. Low-pass Butterworth filtering is commonly used in biomechanical signal processing to attenuate high-frequency noise in kinematic data, with the selected cut-off frequency depending on the movement task, sampling frequency, and signal characteristics [19,20].

Joint-angle baselines were defined separately for each participant, side, and angular variable. When a complete static reference acquisition was available, the baseline corresponded to the mean model-derived joint angle across the valid frames of that acquisition. If a complete static reference acquisition was unavailable, the baseline was calculated as the mean of the first 120 frames of the corresponding trial, representing the initial quiet period. The baseline value was then subtracted from the corresponding model-derived joint-angle time series so that the analysed values represented angular displacement relative to the anatomical reference position.

### 2.4. Joint Angle Computation

Custom local coordinate systems were defined for the trunk, upper arm, forearm, and hand segments. Joint centres were estimated using anatomical definitions based on the midpoint between paired markers when applicable.

For the trunk, the longitudinal axis was defined from MidPelvis to MidShoulder. The mediolateral axis was defined by the vector connecting RAC to LAC, and the anteroposterior axis was obtained via cross-product.

Because midpoint-based reference points are derived from anatomical marker positions, they may be influenced by individual postural characteristics or asymmetries. Therefore, careful and consistent marker placement, together with static reference acquisitions, was used to minimise the influence of postural variability on segment coordinate system definition.

Segment-specific local coordinate systems were constructed using longitudinal and mediolateral anatomical directions. For shoulder-angle computation, the upper-arm direction was defined from the elbow joint centre to the corresponding acromion marker. For elbow- and wrist-related computations, the upper-arm longitudinal axis was defined from the acromion marker to the elbow joint centre. The forearm longitudinal axis was defined from the wrist joint centre to the elbow joint centre, and the hand longitudinal axis was defined from the hand joint centre to the wrist joint centre. Mediolateral directions were defined using the paired medial and lateral epicondyle markers of the humerus for the upper arm, the radial and ulnar styloid markers for the forearm, and the medial and lateral hand markers for the hand. The remaining orthogonal axes were computed using cross-products to form segment-specific local coordinate systems. Axis directions were maintained consistently within each joint-specific calculation, and bilateral sign adjustments were applied to facilitate equivalent interpretation of right- and left-side movements.

**Figure 4:**
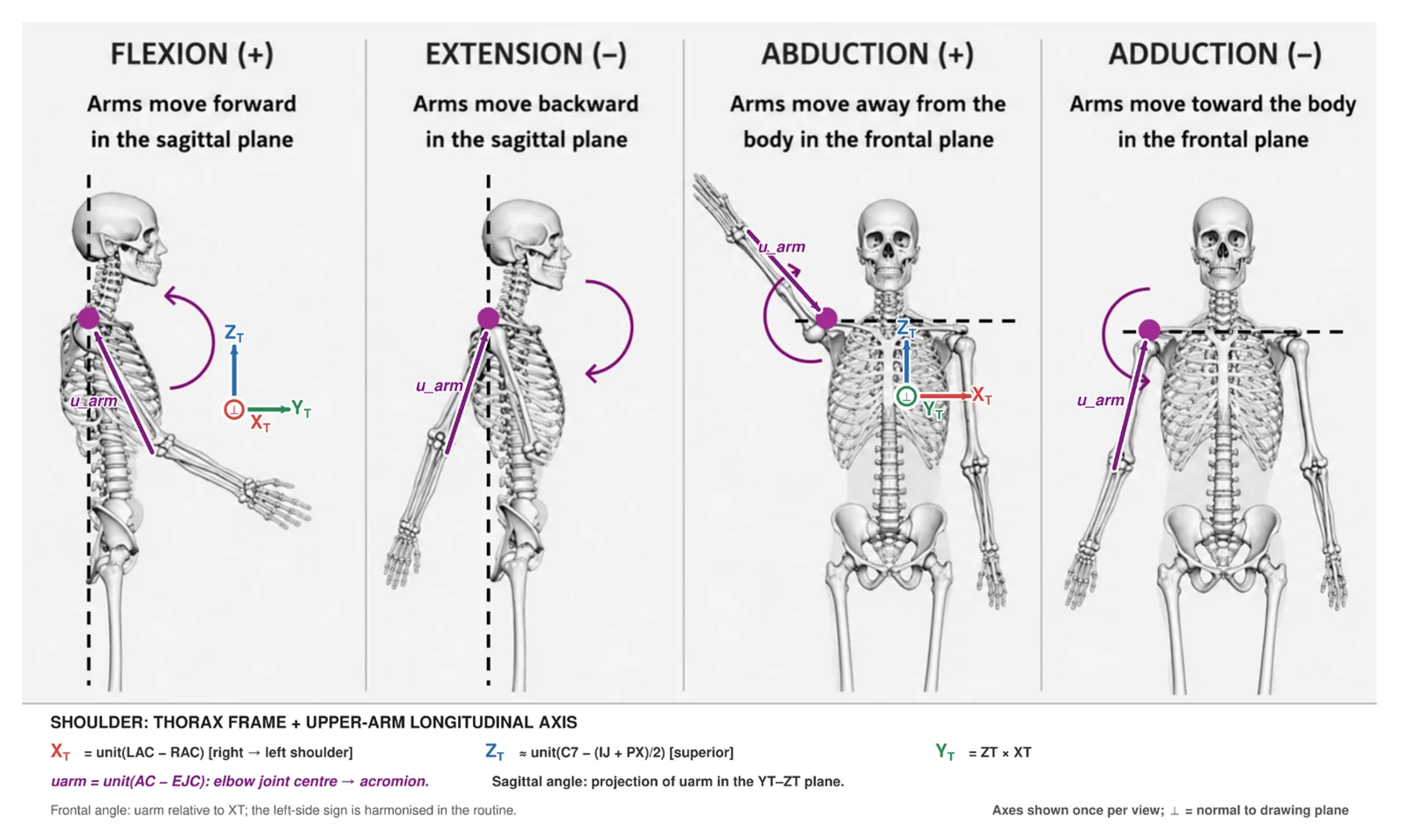
Schematic representation of shoulder joint movements and sign convention. Flexion (+) and extension (−) were defined in the sagittal plane, corresponding to anterior and posterior arm motion, respectively. Abduction (+) and adduction (−) were defined in the frontal plane, corresponding to lateral and medial arm displacement. Movement directions are indicated according to the adopted coordinate system. The local axes shown represent the trunk and upper-arm coordinate systems used for shoulder-angle computation.

Shoulder flexion/extension and abduction/adduction were calculated based on the orientation of the upper arm relative to the trunk coordinate system, as illustrated in Figure 4. Flexion/extension was defined in the sagittal plane, while abduction/adduction was defined in the frontal plane.

**Figure 5:**
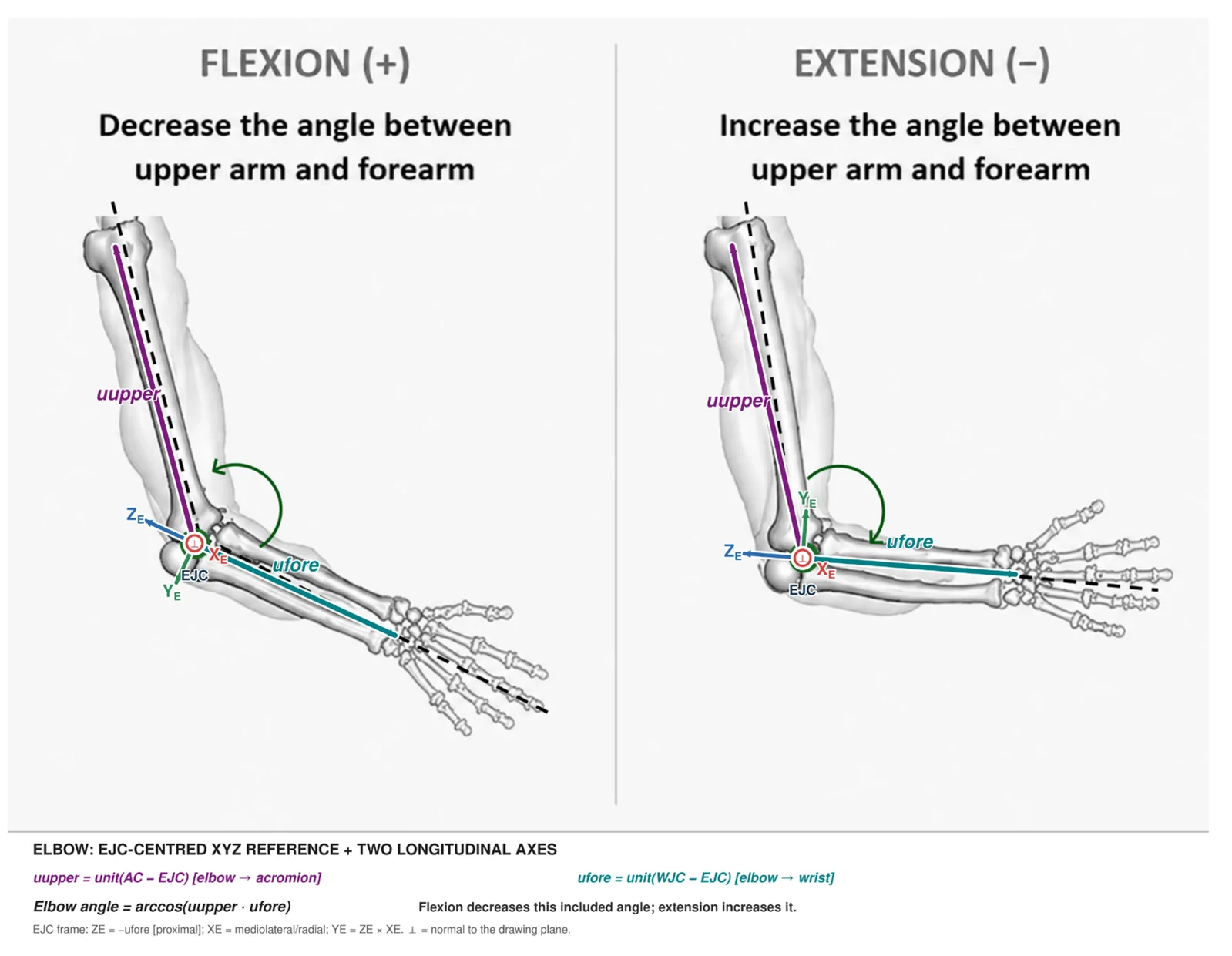
Schematic representation of elbow flexion and extension. Flexion (+) was defined as a decrease in the angle between the longitudinal axes of the upper arm and forearm, while extension (−) corresponds to an increase in this angle. Movement directions follow the adopted coordinate system and sign convention. The local axes shown represent the upper-arm and forearm coordinate systems used for elbow-angle computation.

Elbow flexion/extension was calculated as the angle between the longitudinal axes of the upper arm and forearm (Figure 5).

**Figure 6:**
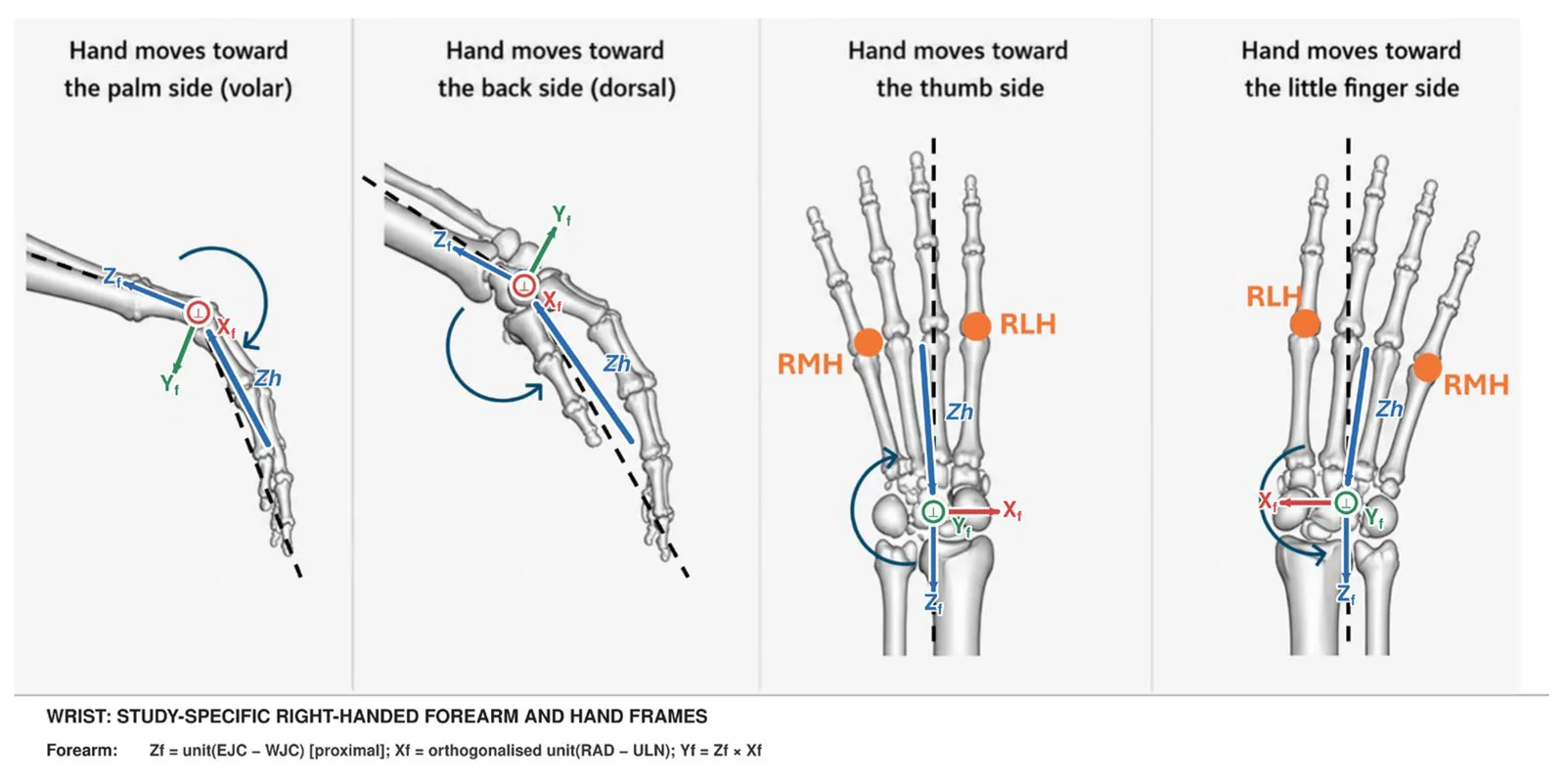
Schematic representation of wrist joint movements. Wrist flexion (+) and extension (−) are defined in the sagittal plane, corresponding to palmar and dorsal motion, respectively. Radial deviation (+) and ulnar deviation (−) are defined in the frontal plane, representing movement toward the thumb and little finger sides. Movement directions are illustrated according to the adopted coordinate system and sign convention. The local axes shown represent the forearm and hand coordinate systems used for wrist-angle computation.

Wrist flexion/extension and radial/ulnar deviation were computed from the relative orientation between the hand and forearm segments, as illustrated in Figure 6. Flexion/extension was defined in the sagittal plane, while radial and ulnar deviation were defined in the frontal plane. Sign conventions were adjusted to ensure consistent interpretation between right and left sides.

Following joint-angle computation, the corresponding participant- and side-specific baseline value was subtracted from each angular time series. Consequently, the reported shoulder, elbow, and wrist values represent angular displacement from the anatomical reference position rather than the uncorrected absolute orientation of the segment coordinate systems.

### 2.5. Technical Evaluation and Data Analysis

The technical feasibility of the proposed methodology was assessed by comparing joint angles obtained from the motion-capture model with the composite reference values derived from the manual and biosignalsplux goniometer measurements during predefined static positions. The evaluated variables corresponded to the main upper-limb joint angles computed by the proposed routines: shoulder flexion/extension, shoulder abduction/adduction, elbow flexion/extension, wrist flexion/extension, and wrist radial/ulnar deviation. Maximum ulnar deviation was included in the acquisition procedure; however, a model-derived angle suitable for quantitative comparison could not be obtained because the marker trajectories required for complete hand and forearm segment reconstruction were unavailable for that condition.

For each joint and movement, participants were positioned in predefined static configurations, including neutral positions and target positions representative of each movement direction. The same positions were recorded with the motion-capture system and assessed using both reference goniometric instruments. For each participant, side, and static position, a composite goniometric reference angle was calculated as the arithmetic mean of the manual and biosignalsplux goniometer measurements. The baseline-corrected model-derived joint angle was then compared with this composite goniometric reference value.

For the motion-capture data, the analysed value corresponded to the mean baseline-corrected joint angle obtained during the stable portion of each static position. The transition frames at the beginning and end of each trial, corresponding to the participant moving into and out of the target posture, were excluded from the analysis. This procedure ensured that the comparison reflected the angular displacement maintained during the static position rather than the transition into or out of the target configuration.

The discrepancy between the motion-capture-derived and goniometric measurements was quantified using absolute error, mean absolute error (MAE), and root mean square error (RMSE), all expressed in degrees. These metrics were selected because they retain direct biomechanical interpretability and remain appropriate for neutral or near-zero reference positions.

The variables assessed during the technical evaluation procedure and the corresponding evaluation methods are summarised in Table 1.

### 2.6. Statistical Analysis

Statistical analysis was descriptive, given the proof-of-concept nature of the study, the inclusion of two participants, and the absence of repeated trials. The analyses were intended to characterise the magnitude of the discrepancies observed in the evaluated static trials and should not be interpreted as population-level estimates of validity or reliability. For each joint, movement, side, and static position, the absolute error between the baseline-corrected model-derived angle and the corresponding composite goniometric reference angle was calculated in degrees.

For each observation, the absolute error was calculated as:

(1) $$Absolute\;error=\;\left|model\;derived\;angle-goniometric\;angle\right|$$

Mean absolute error was calculated as:

(2) $$AE=\frac{1}{n}\sum \left|\hat{y_{i}}-y_{i}\right|$$

Root mean square error was calculated as:

(3) $$RMSE=\;\sqrt{\frac{1}{n}\sum {\left(\hat{y_{i}}-y_{i}\right)}^{2}}$$

where $\hat{y_{i}}$ represents the motion-capture-derived joint angle, y_i_ represents the corresponding composite goniometric reference angle, calculated as the arithmetic mean of the manual and biosignalsplux measurements, and n represents the number of observations included in the relevant summary.

In these equations, the model-derived value represents the baseline-corrected joint angle, while the goniometric reference value represents the arithmetic mean of the two goniometric measurements obtained for the corresponding static position.

Absolute error was calculated for each individual observation. For each joint, movement, static position, and side, MAE and RMSE were calculated across two participant-specific observations, one from each participant. Right- and left-side errors were analysed separately and were not pooled. Descriptive statistics were used to summarise the correspondence between the motion-capture-derived angles and the composite goniometric reference measurements. Because each position was recorded once per participant, no within-session, between-session, or marker-reapplication repeatability metrics were calculated. No inferential statistical comparisons were performed because the study was designed as a controlled proof-of-concept evaluation rather than a group-level hypothesis test.

## 3. Results

### 3.1. Overall Agreement

The comparison between the baseline-corrected motion-capture-derived joint angles and the composite goniometric reference angles showed absolute discrepancies that varied across the evaluated upper-limb positions (Table 2).

Side-specific MAE values ranged from 0.80° to 5.24°, while RMSE values ranged from 0.86° to 5.24°. The largest discrepancies were observed during maximum wrist extension, with an MAE of 3.83° and an RMSE of 4.83° for the right side, and an MAE and RMSE of 5.24° for the left side. For the remaining positions, MAE values were generally below 3°, except for left maximum radial deviation, for which the MAE and RMSE were both 4.10°.

### 3.2. Joint-Specific Results

Wrist flexion/extension showed close correspondence between the baseline-corrected motion-capture-derived angles and the composite goniometric reference angles in the neutral and maximum-flexion positions. Larger discrepancies were observed during maximum wrist extension. The maximum radial-deviation condition showed a larger discrepancy on the left side than on the right side.

Elbow flexion/extension showed MAE values ranging from 0.86° to 2.85° and RMSE values ranging from 1.14° to 3.09°. The smallest discrepancies were observed during maximum flexion, whereas the largest right-side discrepancy was observed in the flexion target position and the largest left-side discrepancy was observed in the neutral position.

Shoulder joint-angle estimates showed MAE values ranging from 0.84° to 2.33° and RMSE values ranging from 1.02° to 2.62°. The largest discrepancies were observed during maximum extension, while neutral, target, and maximum-flexion positions generally showed MAE values below 2°.

### 3.3. Summary of Findings

Overall, the evaluated static observations showed close correspondence between the baseline-corrected model-derived angles and the composite goniometric reference measurements in several positions. These findings provide preliminary support for the technical feasibility of the computational workflow within the restricted scope of the present proof-of-concept evaluation.

## 4. Discussion

The primary aim of this study was to propose and technically evaluate a structured and reproducible methodology for defining upper-limb segments and computing joint kinematics using marker-based motion capture data. The results showed close correspondence between baseline-corrected model-derived joint angles and composite goniometric reference measurements in several evaluated static positions, providing preliminary support for the technical feasibility of the proposed framework under the evaluated conditions.

Marker-based motion capture remains a reference approach for three-dimensional kinematic analysis, including in recent validation studies comparing emerging markerless systems with laboratory-based motion capture. However, its accuracy is strongly influenced by methodological choices related to marker placement, anatomical landmark identification, segment definition, and coordinate system construction [4,5,6,8,9]. In the present study, the magnitude of the observed discrepancies varied according to the joint and static position and should be interpreted in light of uncertainty associated with both reference goniometry and marker-based segment modelling.

A key contribution of this study lies in the explicit definition of segment coordinate systems and joint centres. While recommendations such as those proposed by the ISB provide guidelines for joint coordinate systems, particularly for the shoulder, elbow, wrist and hand, their practical implementation must still be explicitly described in applied studies to ensure reproducibility [10]. This lack of detail can limit reproducibility and lead to inconsistencies across datasets.

Joint-specific results further highlight the influence of anatomical and methodological factors on kinematic estimation. Elbow flexion/extension showed relatively small discrepancies across the evaluated static positions, although the elbow did not consistently demonstrate lower errors than the shoulder or wrist. The largest discrepancies were observed in selected wrist positions, particularly during maximum wrist extension and left maximum radial deviation. These findings may reflect the greater sensitivity of distal and relatively small anatomical segments to marker placement, anatomical landmark identification, soft-tissue artefact, and small differences in segment orientation [6,8,9].

Shoulder discrepancies remained relatively small across most evaluated positions, with the largest values observed during maximum extension. The interpretation of shoulder angles nevertheless remains sensitive to the definition of the trunk reference frame and to the multi-planar nature of shoulder motion. As highlighted in the literature, even small inconsistencies in reference frame definition can significantly influence joint angle estimation, particularly at the shoulder, where multi-planar motion and trunk reference frame definition strongly affect the interpretation of joint angles [4,5,10].

The larger discrepancies observed in selected end-range positions, particularly maximum wrist extension, may be influenced by known sources of uncertainty in marker-based motion capture. At the limits of joint range of motion, soft tissue artefacts and alignment errors can have a greater impact on segment orientation estimation [7,8].

A major strength of this study is the integration of the complete analytical workflow within a transparent and customisable implementation. By explicitly defining marker placement, joint-centre estimation, segment coordinate systems, bilateral sign conventions, trajectory reconstruction, filtering, baseline definition, and joint-angle computation, the framework makes the methodological assumptions traceable and facilitates reproducibility across custom motion-analysis applications.

Although the computational workflow was designed with subsequent dynamic applications in mind, the present study did not evaluate its performance during dynamic, combined, or multi-planar upper-limb tasks. Recent studies comparing marker-based and markerless systems during simulated basketball throwing tasks further highlight the need for transparent and well-described kinematic models when analysing complex sport-specific movements [21].

The present findings should be interpreted within the scope of the study design. The framework was evaluated in two participants using single recordings of controlled static positions, and therefore the study did not examine population-level anatomical variability, repeated marker placement, test–retest repeatability, or performance during continuous combined multi-planar movement. Rotations about the longitudinal axes of the upper arm and forearm were not independently evaluated. Marker placement, reference goniometry, and individual postural characteristics may also have contributed to the observed between-method differences. The use of participant- and side-specific baselines reduced the influence of constant angular offsets in the model-derived joint angles. However, baseline correction does not eliminate errors associated with segment orientation, anatomical landmark identification, marker placement, or goniometric alignment. The observed correspondence should therefore be interpreted as a comparison between baseline-corrected model-derived angles and the composite goniometric measurements obtained for the corresponding static positions, rather than as evidence of agreement in absolute segment orientation. The reconstruction of incomplete marker trajectories may also have introduced uncertainty, particularly for residual missing samples completed after the segment-informed reconstruction stage. Although nearest-neighbour filling was not used as the primary method for defining paired-marker geometry, locally constant trajectory segments may still influence segment orientation estimates. Because the composite goniometric value was itself derived from two measurement procedures with their own alignment and measurement uncertainty, the observed differences should not be attributed exclusively to the motion-capture model. These limitations constrain the interpretation and generalisability of the observed agreement but do not alter the value of providing an explicit and traceable computational workflow. They also identify the next steps required to evaluate the framework in larger samples, across repeated marker applications and sessions, and during dynamic three-dimensional tasks.

## 5. Conclusions

The present proof-of-concept study describes and technically evaluates a structured and reproducible framework for upper-limb segment definition and joint-angle computation using marker-based motion-capture data. The correspondence observed between baseline-corrected model-derived joint angles and composite goniometric reference measurements provides preliminary support for the technical feasibility of the approach under the evaluated controlled static conditions. A key contribution of the framework is the explicit integration of anatomical landmark definitions, joint-centre estimation, segment coordinate systems, trajectory reconstruction, filtering, baseline definition, and joint-angle calculations within a transparent and customisable workflow. Further studies should extend this evaluation to larger samples, repeated acquisitions, and dynamic multi-planar tasks.

## Figures and Tables

**Table 1:** Variables, static positions, reference measurements, and outcome measures included in the technical evaluation.

Joint	Variable	Static Positions Evaluated	Reference Measurement	Outcome Measures
Shoulder	Flexion/ Extension	Neutral Flexion target position Maximum Flexion Maximum Extension	Manual and biosignalsplux goniometers	Baseline-corrected motion-capture-derived angle Composite goniometric reference angle Absolute error Mean Absolute Error (MAE) Root Mean Square Error (RMSE)
	Abduction/ Adduction	Neutral Abduction target position	Manual and biosignalsplux goniometers	
Elbow	Flexion/ Extension	Neutral Flexion target position Maximum Flexion	Manual and biosignalsplux goniometers	Baseline-corrected motion-capture-derived angle Composite goniometric reference angle Absolute error Mean Absolute Error (MAE) Root Mean Square Error (RMSE)
Wrist	Flexion/ Extension	Neutral Maximum Flexion Maximum Extension	Manual and biosignalsplux goniometers	Baseline-corrected motion-capture-derived angle Composite goniometric reference angle Absolute error Mean Absolute Error (MAE) Root Mean Square Error (RMSE)
	Radial/Ulnar Deviation	Neutral Maximum Radial Deviation	Manual and biosignalsplux goniometers	

Note: Maximum ulnar deviation was included in the acquisition protocol but was not included in the quantitative comparison because a complete model-derived angle was unavailable for this condition.

**Table 2:** Side-specific Mean Absolute Error (MAE) and Root Mean Square Error (RMSE), expressed in degrees, between baseline-corrected motion-capture-derived joint angles and composite goniometric reference angles across the evaluated static positions. Each side-specific estimate was based on two participant-specific observations, one from each participant.

Joint	Static Position	Right Side		Left Side	
		MAE(°)	RMSE (°)	MAE(°)	RMSE (°)
Shoulder	Neutral Position (Flex/Ext)	1.14	1.14	1.52	1.54
	Flexion (90°)	1.25	1.47	1.25	1.57
	Maximum Flexion	0.96	1.03	0.84	1.02
	Maximum Extension	2.33	2.62	2.26	2.62
	Neutral Position (Abd/Add)	1.48	1.48	0.86	1.11
	Abduction (90°)	1.54	1.65	1.16	1.29
Elbow	Neutral Position (Flex/Ext)	1.93	2.25	2.60	3.09
	Flexion (90°)	2.85	2.88	2.10	2.19
	Maximum Flexion	0.86	1.14	1.72	2.34
Wrist	Neutral Position (Flex/Ext)	0.93	0.93	2.00	2.00
	Maximum Flexion	0.97	1.05	0.80	0.86
	Maximum Extension	3.83	4.83	5.24	5.24
	Neutral Position (Radial/ Ulnar)	1.00	1.00	2.00	2.00
	Maximum Radial Deviation	1.09	1.09	4.10	4.10

Note: MAE, Mean Absolute Error; RMSE, Root Mean Square Error. Right and left sides were analysed separately and were not pooled. The composite goniometric reference angle was calculated as the arithmetic mean of the manual and biosignalsplux goniometer measurements obtained for the corresponding static position. Two participant-specific observations were included in each side-specific calculation. Maximum ulnar deviation was included in the acquisition protocol but is not reported because a complete model-derived angle suitable for quantitative comparison was unavailable for that condition.

## Data Availability

The data supporting the findings of this study are available from the corresponding author upon reasonable request, subject to ethical and institutional restrictions.

## Abbreviations

The following abbreviations are used in this manuscript:

ISB	International Society of Biomechanics
IOR	Instituto Ortopedico Rizzoli
JC	Joint Centre
RAC	Right Acromion
LAC	Left Acromion
RASIS	Right Anterior Superior Iliac Spine
LASIS	Left Anterior Superior Iliac Spine
RLELB	Right Lateral Epicondyle of the Humerus
LLELB	Left Lateral Epicondyle of the Humerus
RMELB	Right Medial Epicondyle of the Humerus
LMELB	Left Medial Epicondyle of the Humerus
RRAD	Right Radial Styloid Process
RULN	Right Ulnar Styloid Process
LRAD	Left Radial Styloid Process
LULN	Left Ulnar Styloid Process
RLH	Right Lateral Hand Marker
RMH	Right Medial Hand Marker
LLH	Left Lateral Hand Marker
LMH	Left Medial Hand Marker
